# Granulomatosis With Polyangiitis: The Complexity of Clinical Manifestations, Therapeutic Challenges, and Complications of a Severe Multisystemic Case

**DOI:** 10.7759/cureus.47031

**Published:** 2023-10-14

**Authors:** Marta Monteiro, Raquel Domingos, Sara Rocha, Inês Miranda

**Affiliations:** 1 Internal Medicine, Hospital Egas Moniz, Lisbon, PRT; 2 Internal Medicine, Hospital Pedro Hispano, Porto, PRT

**Keywords:** vasculitis, corticotherapy, cyclophosphamide, klebsiella pneumoniae, influenza, renal dysfunction, anti-pr3, anca, granulomatosis with polyangiitis

## Abstract

We report a case of a 34-year-old male with severe multisystemic involvement (including the testis, musculoskeletal system, skin, upper respiratory tract, ocular system, peripheral nerves, abdomen, and kidney) due to granulomatosis with polyangiitis (GPA) and a high proteinase 3 (PR3)-antineutrophil cytoplasmic antibodies (PR3ANCA) titer. A renal biopsy showed pauci-immune glomerulonephritis (GN). Systemic corticotherapy combined with cyclophosphamide was chosen for induction therapy. During the induction phase, clinical deterioration occurred in the form of severe alveolar hemorrhage, leading to admission to the intensive care unit (ICU). Influenza A (H1N1) was detected in the respiratory tract. Furthermore, blood sampling revealed an invasive *Klebsiella pneumoniae* infection that persisted despite multiple antibiotic regimens. A CT scan showed splenic vascular compromise, assumed to be the primary source of the infection, with sustained improvement after splenectomy. Maintenance therapy included a tapering dose of corticotherapy for 36 months and azathioprine 100mg daily for five years, which achieved full and sustained remission. The patient has been in full remission for nine years, with mild renal sequelae, including proteinuria and secondary hypertension.

## Introduction

Antineutrophil cytoplasmic antibodies (ANCA)-associated vasculitis forms a complex group of diseases. Their interest lies not only in the multisystemic range of signs and symptoms but also in the multiplicity of differential diagnoses, making a complete analysis of the patient and a multidisciplinary approach mandatory in clinical practice.

Granulomatosis with polyangiitis (GPA) is an entity within this group, with its classic presentation being inflammatory necrotizing granuloma formation in the respiratory tract as well as small vessel necrotizing vasculitis [1]. The most frequent sites of involvement are the upper and lower respiratory tracts, kidneys, and eyes. Less frequently affected sites include the skin, nervous system, joints, heart, and gastrointestinal tract. Urogenital manifestations are a rare form of the disease [2].

The key to the diagnosis relies on the constellation of clinical findings, as the absence of ANCA against serine proteinase 3 (PR3) or its histological hallmark, the inflammatory granulomas, does not exclude it [1]. We present a case with severe multisystemic involvement, including urogenital involvement (orchitis), which is reported in less than 1% of studies with a large number of patients [2].

## Case presentation

A previously healthy 34-year-old man presented with a two-month constitutional syndrome characterized by asthenia, anorexia, and weight loss (quantified at 8% of total body weight). He also reported diffuse abdominal pain and self-limited left gonalgia associated with functional impairment. After a few weeks, persistent asthenia and the reappearance of arthralgias in the knees and wrists with an inflammatory pattern were documented. In addition, he experienced a productive mucopurulent cough, nasal congestion, and sanguinolent rhinorrhea, as well as inguinal and left testis pain.

Upon observation, fever, bilateral conjunctival hyperemia, a maculopapular rash, oral ulceration, and testicular tenderness were found. Blood work revealed leukocytosis (20,700 U/L), neutrophilia (15,560 U/L) with mildly elevated eosinophil counts (560 U/L), high inflammatory markers (lactate dehydrogenase (LDH): 959 U/L and C-reactive protein (CRP): 18 mg/dL), a hepatocellular cytolytic pattern, and normal kidney function (creatinine: 1.06 mg/dL). Urine analysis showed proteinuria, hemoglobinuria, and the presence of urobilinogen. He was then admitted for further investigation and treatment.

Investigations

A multidisciplinary approach was ensured with evaluations from various specialties. Dermatology assessed the rash, which was found to be nonspecific and not eligible for biopsy. Ophthalmology diagnosed bilateral conjunctivitis and anterior uveitis.

**Figure 1:**
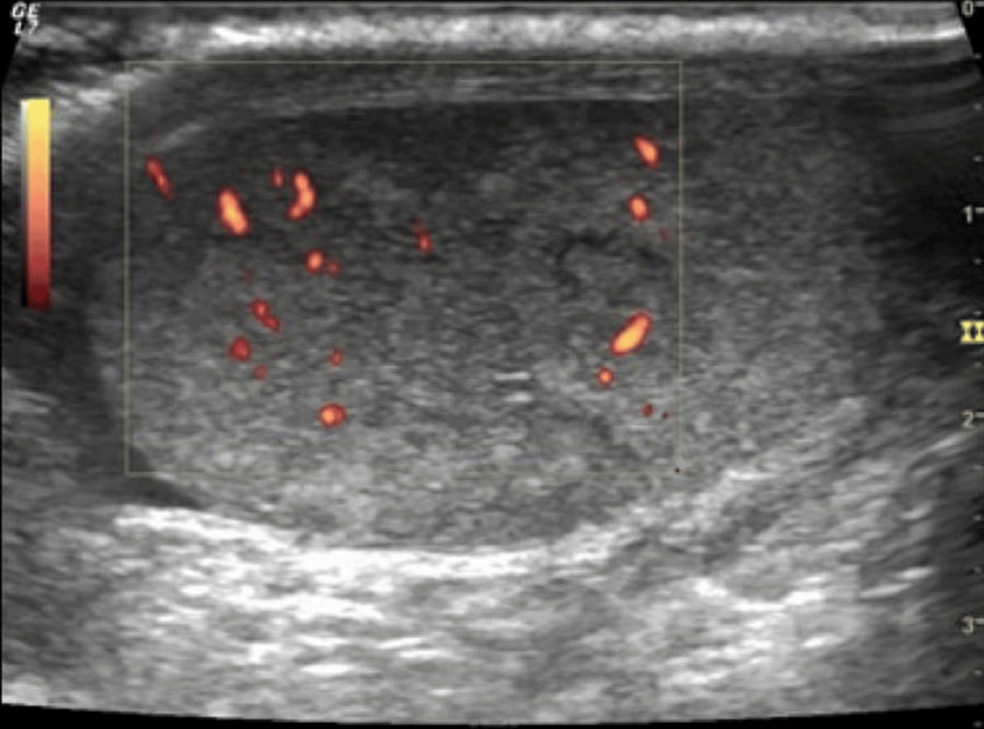
Scrotal ultrasound Heterogeneity in the left testis, with pseudo-nodular areas

A scrotal ultrasound revealed heterogeneity in the left testis, with pseudo-nodular areas (Figure 1).

No changes in morphology or vascular territory were noticed. Considering the symptoms of left testicular pain and tenderness, along with the absence of nodules or inflammatory signs, the findings were indicative of orchitis.

Throughout the hospitalization, the patient's renal function rapidly deteriorated, with oliguria and a creatinine level that reached a maximum of 3.8 mg/dL. Due to the initial findings of the summary urine analysis, a urinary sediment evaluation was performed, revealing dysmorphic red blood cells (RBCs) in two out of three samples, suggesting rapidly progressive glomerulonephritis [3].

The 24-hour urine analysis showed a total volume of 550 mL and subnephrotic proteinuria, with a significant contribution of albumin (total proteins: 1524.1 mg/24h (reference range: <140) and albumin: 407 mg/24h (reference range: <30)), resulting in an albumin/protein ratio of 0.26. The renal ultrasound revealed only nonspecific findings of nephropathy, including an increase in renal diameter and loss of parenchymal differentiation.

The observation by the otorhinolaryngologist revealed the presence of rhinitis with exclusive involvement of the nasal fossa, with no changes in the perinasal sinus. Septal mucosa atrophy with a high risk of perforation was present, and the results of biopsy samples showed nonspecific findings (intense mixed inflammatory infiltrate, including lymphocytes, plasmacytes, neutrophils, eosinophils, and some giant Langerhans cells), which were only available after treatment initiation.

Computed tomography scans of the mastoid, perinasal sinus, and orbits were performed and showed no pathological findings. Hand and wrist X-rays did not reveal deformities or erosive changes.

Additionally, during the hospital stay, paresthesia on the external aspect of the right leg developed. Electromyography showed a sensitive axonal mononeuritis of the right external saphenous nerve. Neurosensory involvement was ruled out after performing tonal and vocal audiograms and a tympanogram.

Differential diagnosis

The patient's general status continued to deteriorate, with an intense constitutional syndrome of multisystemic affection characterized by persistently high CRP levels and an LDH value that reached 1300 U/L. A diagnosis was essential, and due to the major role of renal dysfunction as well as its rapid progression, the diagnostic workup was centered around it.

Three categories of possible etiologies were considered: infectious, neoplastic, and immune-mediated. All tested serologies were negative (human immunodeficiency virus (HIV), hepatitis B virus, hepatitis C virus, *Treponema pallidum*, cytomegalovirus, Epstein-Barr virus, *Chlamydia pneumoniae*, *Mycoplasma pneumoniae*, and *Toxoplasma gondii*), as well as microbiological testing of urine and blood. Endocarditis was less likely due to the absence of cardiac symptoms and heart murmurs.

A full-body CT scan was performed and showed no evidence of a malignant process or pulmonary embolism. There were no nodules, cavitations, consolidations, or any other type of pulmonary involvement.

The immunological evaluation showed a positive c-ANCA, anti-PR3, with a high titer (6700 UA/mL), making the diagnosis of GPA the most likely. Approximately 90% of GPA patients have ANCA-positive antibodies, and among those, 70%-80% are against PR3 [4]. The predictive value of ANCA antibodies in the diagnosis of GPA strongly correlates with the clinical presentation. It is known that in a patient presenting with rapidly progressive glomerulonephritis (GN), the precision of the diagnosis increases to 98% [5]. Other markers for immunomodulated glomerular disease did not reveal any changes (quantitative analysis of immunoglobulins; antibodies like antinuclear antibodies (ANAs), Anti-double-stranded deoxyribonucleic acid (anti-dsDNA) antibodies, anti-glomerular basement membrane (anti-GBM); cryoglobulins were negative).

The Birmingham score was used to evaluate the activity of GPA. Although mainly applied in clinical trials and scientific research, it can be of clinical value. It is known that a relapse of the disease may occur in a previously unaffected organ, and a structured evaluation of multisystemic involvement may be useful [6]. At the time of diagnosis, the patient had a score of 38. The Birmingham scale ranges from zero points, corresponding to total remission or absence of evidence of activity in the last 28 days, to a maximum of 68 points [7].

Treatment

Treatment of GPA is divided into two stages: the induction phase, which aims for full remission, and the maintenance phase, which aims to avoid relapses. Pharmacological options for the induction phase include systemic cyclophosphamide and rituximab [6]. For the maintenance phase, options include methotrexate, azathioprine, and rituximab, with treatment duration ranging from 24 months to indefinitely [6]. The choice of agent is tailored based on kidney and/or liver involvement, and the superiority of any specific agent remains unclear.

When severe organ damage manifestations exist, the induction regimen should include systemic corticosteroids in combination with an immunosuppressive agent [6]. Whether it is cyclophosphamide or rituximab, the combination regimen induces remission in 75%-90% of patients, with 50%-70% experiencing complete remission between three and six months [8].

Starting treatment without a biopsy of an affected site remains controversial [1,6]. If it cannot be obtained immediately, treatment should not be delayed, but the biopsy should be performed as soon as possible, even after the initiation of therapy. At this time, the results of the nasal biopsy remained unavailable. Daily contact with nephrology was made due to the imminent need for renal replacement therapy, but the renal biopsy was not performed due to the frailty of the patient and the high risk of complications.

Due to the severity of the illness, treatment was initiated with intravenous cyclophosphamide (1 g, corresponding to a dose of 15 mg/kg), with a total of two in-hospital administrations, and with methylprednisolone (1 g/daily, for three days), transitioning to prednisolone on the fourth day (1 mg/kg/day). Five days after the initiation of treatment, the clinical scenario began to improve, with apyrexia, a reduction in inflammatory markers (CRP, LDH), and a favorable evolution of knee arthritis and eye involvement. Creatinine levels initially rose to 6.5 mg/dL but then started to normalize.

To minimize the risk of infectious complications due to immunosuppression, latent tuberculosis infection was excluded. Other measures included a stomatology evaluation for oral hygiene and scaling, as well as sperm collection due to the risk of infertility associated with cyclophosphamide [6]. Pharmacological prophylaxis of pneumocystosis with trimethoprim and sulfamethoxazole [6], ulcer prevention with a proton pump inhibitor, and supplementation with folinic acid, cholecalciferol, and calcium were started.

The nasal biopsy result became available at this point, showing an ulcerated nasal mucosa filled with an intense inflammatory lymphoplasmacytic infiltrate rich in neutrophils, eosinophils, and Langhans cells. No vasculitic features were found. Although not conclusive, it could be consistent with the GPA diagnosis. Considering the sustained improvement, the patient was discharged and referred to an internal medicine appointment for follow-up.

Outcome and follow-up

After discharge, systemic corticotherapy with prednisolone (1 mg/kg/day) was maintained, as well as the above-mentioned prophylaxis and supplementation. Two additional administrations of cyclophosphamide were given at week four and week seven.

**Figure 2:**
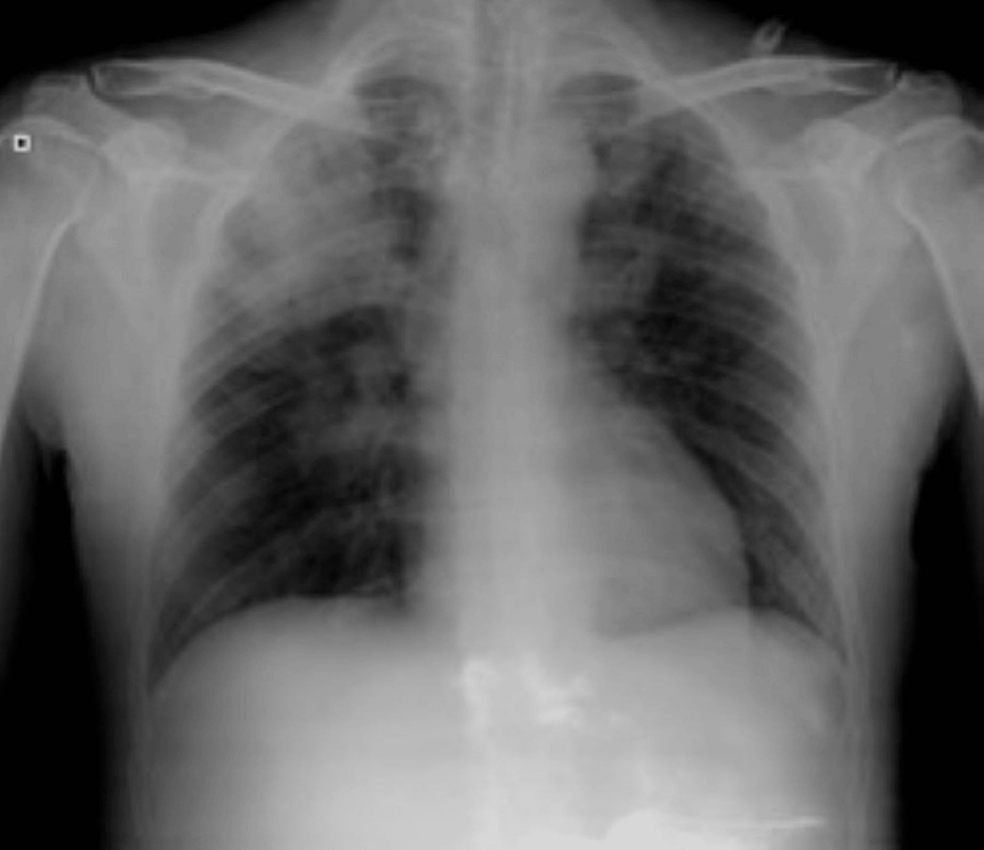
Chest X-ray Bilateral infiltrates in the upper pulmonary fields

At this time, the patient described a productive cough with a three-day evolution that worsened overnight. He also reported fever, fatigue, shortness of breath, and hemoptysis sputum, which led him to the hospital emergency department. Upon admission, pallor, dehydration, tachypnea with supplemental oxygen of 4 L/min, and bronchospasm dominated the clinical picture. Blood work revealed anemia of 8.6 g/dL, leukocytosis of 11500 U/L with 81.2% of neutrophilia, and mild thrombocytopenia (125,000 platelets). The renal function had improved compared to the time of discharge (creatinine: 3.76 mg/dL), but urine analysis still showed hemato-proteinuria. The PR3-ANCA titer was substantially lower than the previous measurement (117 UA/mL). High inflammatory markers stood out, with an LDH of 1179 U/L and a CRP of 5.73 mg/dL. Arterial gases revealed hypoxemia of 67 mmHg and hypocapnia of 30.6 mmHg. A chest X-ray showed bilateral infiltrates (Figure 2).

**Figure 3:**
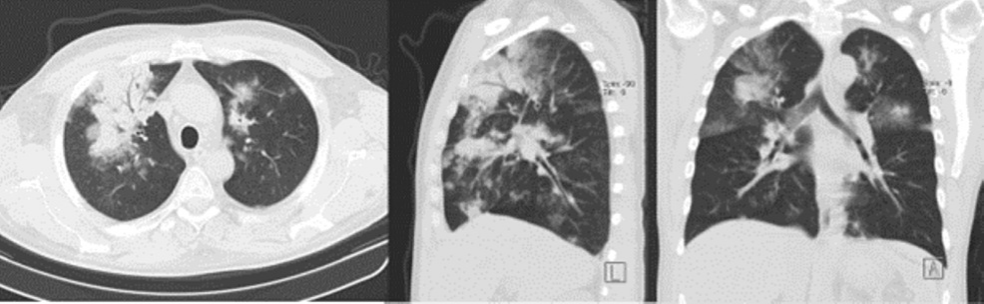
Chest CT scan Diffuse ground glass opacities and areas of consolidation with an air bronchogram in the medium right lobule and lower left lobule

To clarify the significance of the predominant left apical infiltrate, in addition to the right para-hilar and apical infiltrates on the chest X-ray, a CT scan was performed. The CT scan showed diffuse ground glass opacities as well as areas of consolidation with an air bronchogram in the medium right lobule and lower left lobule, which were compatible with alveolar hemorrhage (Figure 3).

These findings lead to ICU admission. Considering the previous diagnosis of GPA, the alveolar hemorrhage raised the dilemma between framing the clinical picture under vasculitic involvement versus infectious complications. In the first few days, given the severity of the clinical picture, the immunosuppressive therapy was intensified with three pulses of methylprednisolone, four sessions of plasmapheresis, and one administration of rituximab. Biological fluid samples were collected (blood, respiratory secretions, bronchoalveolar lavage, urine, and nasal exudate for viruses), and tuberculosis prophylaxis with isoniazid was started.

The patient's condition acutely worsened, evolving into acute respiratory distress syndrome (ARDS), requiring endotracheal intubation for mechanical ventilation, deep sedation, and vasopressor support, complicated by renal failure necessitating renal replacement therapy. Simultaneously, influenza A (H1N1) viruses were identified on a nasopharyngeal swab, and therapy with oseltamivir was instituted for 14 days.

On the twentieth day of the ICU stay, the worsening of respiratory function led to the collection of respiratory and blood samples, revealing the isolation of *Klebsiella pneumoniae* in both sites. A total course of 31 days of directed antibiotic therapy was carried out (19 days of meropenem, eight days of colistin, and four days of amikacin) due to the persistence of bacteremia with a similar antimicrobial susceptibility profile. Endocarditis was excluded with normal transthoracic and transesophageal echocardiograms.

**Figure 4:**
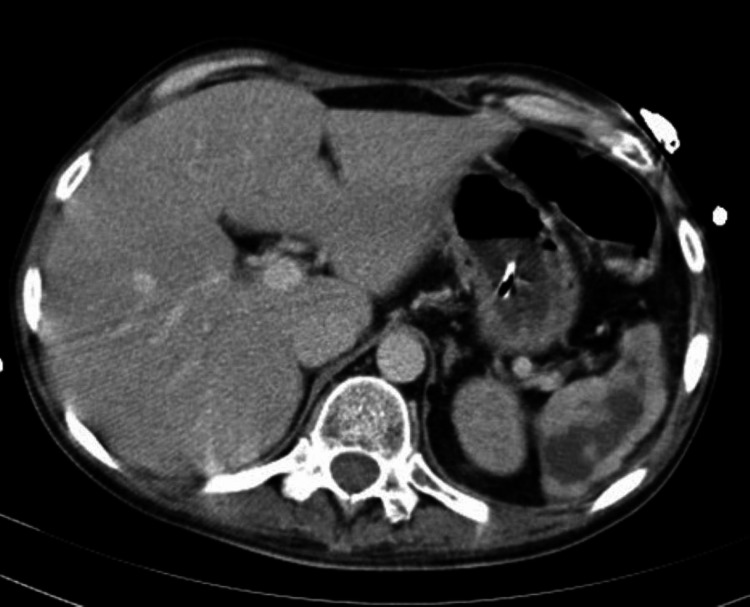
Abdominal cut of the full body CT scan Reveals the presence of nonspecific splenic heterogeneity that could be related to vascular compromise

A full-body CT scan was performed to search for the source of infection, revealing the presence of nonspecific splenic heterogeneity that could be related to vascular compromise (Figure 4).

A CT scan is very sensitive for detecting lesions in the spleen, with a limited number of autopsy studies showing a high percentage (78%-100%) in GPA patients [9]. Splenic infarction can lead to complications such as functional hyposplenism, which has been linked to an increased risk of developing serious infections. This risk increases with the use of immunosuppressive drugs [9].

**Figure 5:**
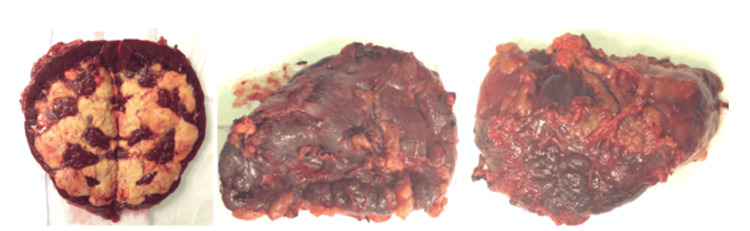
Surgical piece Hemorrhagic necrosis and deposits of amyloid substance

Considering these findings, the spleen was assumed to be the source of the infection, and a surgical splenectomy was performed (Figure 5).

From this point onwards, a clear clinical and analytical improvement was observed with sterile blood samples, a reduction in inflammatory markers, hemodynamic stabilization, and suspension of multiorgan support. The histological analysis revealed hemorrhagic necrosis, with no evidence of vasculitis or granulomas but mild perivascular deposits of amyloid substance and signs of extramedullary hematopoiesis. During the same surgical procedure, a renal biopsy by laparotomy was performed, collecting a large sample of 100 glomeruli. The results revealed pauci-immune GN in 60% of crescents, reinforcing the diagnosis of GPA [1].

After a two-month stay in the ICU, finally reaching clinical stabilization, the maintenance phase of therapy was initiated with a reduction in systemic corticosteroid therapy (prednisolone 20 mg/day) and titration of the azathioprine dose up to 2 mg/kg/day. Physiatry and nutritional evaluations were ensured, and an intensive physical rehabilitation program due to ICU myopathy as well as a dietary plan to reverse malnutrition were designed. At the time of discharge, the patient could walk short distances, renal function was nearly normal (creatinine: 1.22 mg/dL), and ANCA titers were persistently negative. Remission criteria were attained.

During ambulatory follow-up, a full recovery was achieved, and maintenance therapy for 36 months was completed. Azathioprine, 100 mg/day, was suspended after five years. To this date, the patient has maintained full remission for nine years, with a score of three points on the Birmingham scale due to mild renal sequelae with proteinuria and secondary hypertension medicated with ramipril 10 mg/day. The ANCA titers remain persistently negative.

## Discussion

Granulomatosis with polyangiitis is a severe autoimmune disease of unknown origin that targets small and medium-sized vessels. It typically presents with multisystemic involvement and has a poor prognosis unless aggressive immunosuppressive therapy is initiated [4]. While environmental, genetic, and infectious triggers have been associated with the disease, they seem insufficient to explain the entire pathogenesis [1]. The hallmark is necrotizing granulomatous inflammation of the upper and lower respiratory tracts with necrotizing vasculitis of small and medium-sized vessels, along with positive ANCA testing, primarily directed against serine PR3 but also myeloperoxidase [1].

According to the latest recommendations, a positive biopsy strongly supports the diagnosis of vasculitis and is recommended to establish a new diagnosis in patients with suspected relapsing vasculitis [6]. Therefore, pauci-immune GN, or necrotizing vasculitis in any organ, remains the gold standard for diagnostic purposes [6].

Renal biopsies reveal pauci-immune GN in up to 91.5% of individuals with GPA, whereas biopsies of the upper respiratory tract more often reveal unspecific findings rather than granulomatous inflammation [6]. Due to the inability to perform a renal biopsy to establish the diagnosis, we had to rely on clinical manifestations and ANCA testing to make the diagnosis and initiate treatment. Remission-induction therapy with a combination of corticoids and pulsed cyclophosphamide was administered, given the moderate to high activity score and multisystemic involvement (testis, musculoskeletal, skin, respiratory, ocular, kidneys, peripheral nerve, abdomen) with rapidly progressive renal disease.

Virtually all organs can be affected, but the respiratory tract and kidneys are by far the most frequently affected [1]. Urogenital involvement is rare, reported in less than 1% of cases, and among those, only up to 36% are related to testicular vasculitis [2]. According to a review on urologic and male genital involvement in GPA, when it is present, it usually occurs as part of a generalized systemic disease with constitutional symptoms, pulmonary involvement (81%-87%), kidney involvement (45%-60%), and upper respiratory tract involvement (90%-100%) [2]. Laboratory findings typically include increased acute phase reactants and ANCA against serine PR3 in 90% of cases [2].

In a series of case reports, a multisystemic phenotype with ocular, articular, and neurological involvement was associated with urogenital manifestations in various forms (orchitis, epididymitis, prostatitis, etc.) as an inaugural sign that preceded the diagnosis of GPA [10]. A considerable tendency to relapse under maintenance therapy was noted, although a high sensitivity to corticotherapy was observed [10].

Less than a month after the initiation of therapy, the patient developed alveolar hemorrhage and hemodynamic compromise, leading to ICU admission. In cases where a relapse of GPA is life-threatening, maintenance therapy should include a combination of corticoids and either cyclophosphamide or rituximab [6]. In this case, all three were administered since the possibility of a relapse could not be completely excluded. Plasma exchange was performed, recommended both for managing the hemorrhage and for addressing the rapidly worsening renal function, with creatinine levels reaching 6.5 mg/dL (over 5.7 mg/dL) [6].

However, the diagnosis turned out to be influenza A (H1N1)-associated alveolar hemorrhage. A retrospective study in India in 2009, which necropsied 15 patients with severe influenza A (H1N1), reported that 80% of the histological findings were compatible with intra-alveolar hemorrhage [11]. Additionally, extensive autopsies during the 1918 pandemic noted diffusely swollen and inflamed bronchial surfaces with evidence of hemorrhagic bronchitis and tracheobronchitis in 50% of the cases, often with a luminal filling of frothy blood-stained material believed to be fibrin and erythrocytes [12].

It should be noted that the frequency of splenic involvement, particularly infarction, may be underestimated in GPA patients, and immunosuppressive therapy is another risk factor for the development of serious infections [9]. The CT scan findings pointing towards vascular compromise in a patient with a metastatic *Klebsiella pneumoniae* infection, which improved after a splenectomy, support this hypothesis. There is no gold standard or demonstrated superiority of antibiotic treatment versus a percutaneous approach or splenectomy in these cases [13].

For maintenance purposes, low-dose corticosteroid therapy and either azathioprine, rituximab, methotrexate, or mycophenolate mofetil are recommended [6]. Once complete remission or sustained improvement (partial remission) is achieved, maintenance therapy can be initiated after two to four years from the last administration of cyclophosphamide [8].

There is no clear evidence regarding the duration of treatment, but early cessation is associated with higher recurrence rates [6]. Maintenance therapy with a combination of agents (prednisolone and azathioprine) was extended for 36 months due to the high risk of relapse associated with higher scores on the Birmingham activity scale and ANCA directed against serine PR3 [4,7]. For the same reason, azathioprine was continued for five years. In a young male with no other comorbidities, minor sequelae, or negative PR3-ANCA and one relapse in the middle of induction therapy, there is no evidence supporting indefinite immunosuppressive therapy over extended therapy [8].

For follow-up purposes, proper immunization with influenza, pneumococcus, and meningococcus vaccines is ensured, as is the evaluation of organ involvement with acute phase reactants, ANCA, renal function, CT scans, and periodic 24-hour ambulatory arterial pressure monitoring for cardiovascular assessment. This evaluation is of utmost importance since GPA patients have a high risk of long-term complications both from the disease and from the treatment, including cardiovascular disease, diabetes, osteoporosis, and malignancy. Approximately one-third of patients have more than five items of damage at a mean of seven years after diagnosis [6]. The most frequent complications include diabetes and hypertension, and our patient is no exception, with renal and iatrogenic hypertension as sequelae. The mortality rate is 2.7 times higher than that of the general population, but if left untreated, it can be as high as 90% [8].

## Conclusions

Granulomatosis with polyangiitis is a multisystemic disease that can affect virtually all organ systems, with the respiratory tract and kidneys being the most frequently affected. The severity and complexity of this case are attributed not only to the manifestations of the disease but also to potentially life-threatening complications that can arise. Distinguishing between disease relapse and refractory disease is challenging, given the limited time since the initiation of remission induction therapy.

Determining the relative contribution of disease activity versus its complications to the clinical presentation remained a significant clinical challenge, particularly since alveolar hemorrhage can be triggered by both GPA and influenza A (H1N1). The morbidity and mortality associated with GPA are greatly influenced by the development of complications. The second hospitalization in this case was marked by both alveolar hemorrhage and a splenic reservoir of an invasive *Klebsiella pneumoniae* infection, necessitating mechanical ventilation, multiple antibiotic regimens, and emergency surgery.
